# Research on Teaching Practice of Blended Higher Education Based on Deep Learning Route

**DOI:** 10.1155/2022/5906335

**Published:** 2022-01-13

**Authors:** Yang Li, Lijing Zhang, Yuan Tian, Wanqiang Qi

**Affiliations:** ^1^Aviation University of Air Force, Changchun, Jilin 130022, China; ^2^School of Automotive Engineering, Jilin Teachers Institute of Engineering and Technology, Changchun, Jilin 130022, China

## Abstract

This paper establishes a hybrid education teaching practice quality evaluation system in colleges and constructs a hybrid teaching quality evaluation model based on a deep belief network. Karl Pearson correlation coefficient and root mean square error (RMSE) indicators are used to measure the closeness and fluctuation between the effective online teaching quality evaluation results evaluated by this method and the actual teaching quality results. The experimental results show the following: (1) As the number of iterations increases, the fitting error of the DBN model decreases significantly. When the number of iterations reaches 20, the fitting error of the DBN model stabilizes and decreases to below 0.01. The experimental results show that the model used in this method has good learning and training performance, and the fitting error is low. (2) The evaluation correlation coefficients are all greater than 0.85, and the root mean square error of the evaluation is less than 0.45, indicating that the evaluation results of this method are similar to the actual evaluation level and have small errors, which can be effectively applied to online teaching quality evaluation in colleges and universities.

## 1. Introduction

In the process of informatization teaching with the in-depth integration of information technology and classrooms, teaching methods tend to be more online integrated with offline. At present, we are in a ubiquitous learning environment supported by mobile Internet; how to effectively integrate combining resources, deep learning promoted by design technology, is a question worth exploring and thinking about. With microclasses, MOOCs (massive open online courses), SPOC (small private online course), and flipped classroom development, blended teaching has gradually become one of the important directions of college teaching reform. Blended teaching complements the advantages of traditional classroom teaching and online learning to achieve the optimization of learning effects [1], which is in line with the purpose of deep learning. Through blended teaching, making full use of high-quality resources and tools, and reconstructing the teaching process, an effective teaching plan can be provided for the realization of deep learning.

The current research is mainly concentrated in the field of higher education. The content of the research includes influencing factors, technical support, application models, strategies, blended teaching and online learning, effects and quality, evaluation, and other issues [2]. Based on the analysis of the relevant literature on online learning and blended teaching, Shivetts proposed that students' learning motivation is the main factor for the success of online learning and blended learning. In a blended learning environment, students' learning results and curriculum settings and accessibility sex is closely related [3]. Graff and Ayden conducted research from the perspective of classroom community awareness [4,5], Aspden conducted research from the perspective of student participation and interaction [6], Oliver and Trigwell passed based on the problem-based learning framework to explore the evaluation of blended teaching and so on [7]. Based on the exploration community theory, Garrison et al. further constructed a specific hybrid teaching evaluation framework [8, 9].

The primary task of developing mixed teaching in colleges and universities is how to improve the quality of online teaching and informatization teaching [10]. Therefore, it is very important to study effective methods for evaluating online teaching quality in colleges and universities. Research on online teaching quality evaluation methods in colleges and universities can strengthen online teaching quality management and improve online teaching quality [11]. A deep belief network is an important learning model in deep learning, formed by combining low-level features and more abstract high-level features or attribute categories. It has received widespread attention from all walks of life and has since set off a wave of deep learning research. In recent years, deep belief networks have been widely used in curriculum areas closely related to people's lives and can realize machine translation, face recognition [12], speech recognition, signal recovery, business recommendation, financial analysis, medical assistance, and intelligent transportation. The deep learning network has good robustness and high accuracy. Based on this, this paper studies the method of evaluating the quality of mixed teaching in colleges and universities so as to improve the quality of mixed teaching in colleges.

## 2. Related Work

### 2.1. Deep Belief Neural Network

The deep belief network (DBN) model is a series of restricted Boltzmann machine (RBM) models stacked from bottom to top, using an unsupervised greedy layer-by-layer method to pretrain multiple RBMs, which means that each layer of RBM needs to be trained. The weight value enables the hidden layer to obtain the connection of the high-level data expressed by the visible layer [13].

#### 2.1.1. Basic Principles

The training process of the deep belief neural network can be summarized into two parts: one is pretraining, which uses unsupervised layer-by-layer learning to initialize the parameters of the neural network structure, and the other is fine-tuning (fine-training). At the end of the model, the backpropagation algorithm is used to fine-tune the network parameters globally. Even if the entire network has accumulated multiple layers, the parameters can still be reasonably optimized. This learning method solves the problem of gradient disappearance and makes the learning of deep neural networks more efficient.

#### 2.1.2. Restricted Boltzmann Machine

**Figure 1:**
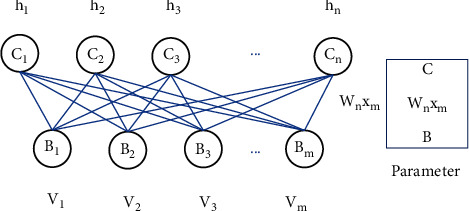
RBM model structure diagram.

Restricted Boltzmann machine (RBM) originated from the Boltzmann machine (BM). BM not only has a strong unsupervised learning ability but also can learn complex rules in data [14], but this kind of training is relatively complicated, and the training time is longer. In order to overcome this problem, Reikard introduced a restricted Boltzmann machine [15], whose structure is shown in Figure 1.

RBM is composed of a visible layer (visible) and a hidden layer (hidden), and there is no connection in the layer. *h*_1_ to *h*_*n*_ are *n* real numbers, V_1_ to *V*_*m*_ are *m* real numbers. These real numbers are all numbers between 0 and 1, and each of them forms an *h* vector and a *v* vector. There is a weight *w* between each explicit layer and the hidden layer, which has a total weight of n·and m, and *c* and *b* are the bias vectors of the hidden and explicit layers, respectively.

RBM is an energy-based model. Then, for a given set of states (*v*, *h*), the energy formula is defined as follows:

(1) $$\begin{array}{c} E\left(v,h|\theta \right)=-\left(\underset{ij}{\sum }w_{ij}v_{i}h_{j}+\underset{i}{\sum }b_{i}v_{i}+\underset{j}{\sum }c_{j}h_{j}\right). \end{array}$$

Among them, *θ* is the parameters *w*, *c*, and *b*. The energy represented on the right side of the equation has three parts. One is generated by the weight *w* connecting the nodes *v* and *h* on both sides, and all three of them must be 1 to be considered as energy output; the other two are the multiplication of the offset on the node and the vector dimension value of the node input, and the same three must be 1 to be considered as energy output. Each energy corresponds to a state; the smaller the energy, the more stable the model. When the parameters are determined, the joint probability distribution of the explicit layer and hidden layer neurons can be obtained from the energy formula as follows:

(2) $$\begin{array}{c} P_{\theta }\left(v,h\right)=\frac{1}{Z\left(\theta \right)}e^{-E\left(v,h|\theta \right)}, \end{array}$$

(3) $$\begin{array}{c} Z\left(\theta \right)=\underset{v,h}{\sum }e^{-E\left(v,h|\theta \right)}. \end{array}$$

Therefore, when determining the state of the input layer, the activation states of the hidden layer nodes are independent of each other. Therefore, the activation probability of the i-th hidden layer node is as follows:

(4) $$\begin{array}{c} P\left(h_{i}=1|v\right)=\sigma \left(\overset{m}{\underset{j=1}{\sum }}w_{ij}\times v_{j}+c_{i}\right), \end{array}$$

*σ*(*x*)=1/1+exp(−*x*) is the activation function (Sigmoid).

Given that the structure of the restricted Boltzmann machine is symmetrical, when determining the state of the hidden layer node, the activation state of each input layer node is also conditionally independent, so in the same way, the activation probability of the j-th visible unit can be expressed as follows:

(5) $$\begin{array}{c} P\left(v_{i}=1|h\right)=\sigma \left(\overset{n}{\underset{j=1}{\sum }}w_{ij}\times h_{i}+b_{i}\right). \end{array}$$

#### 2.1.3. Algorithm Execution Process of the Restricted Boltzmann Machine

In 2002, Brosch proposed a fast-learning algorithm for RBM-Contrastive Divergence (CD) [16]. In the CD algorithm, the state of the visible layer node can be used as the data feature value, and the activation state of all hidden layer nodes is calculated by formula (4). After the states of all hidden layer nodes are determined, the probability is calculated that the value of the i-th input layer node iv is 1 according to formula (5), and then, a reconstruction of the visible layer is obtained. In this way, when using the value of the log-likelihood function of the stochastic gradient ascent method on the training data, the update criterion of each parameter is as follows:

(6) $$\begin{array}{c} \Delta W_{ij}=e\left(<v_{i}h_{j}{>}_{\text{data}}-<v_{i}h_{j}{>}_{\text{recon}}\right), \end{array}$$

(7) $$\begin{array}{c} \Delta b_{i}=e\left(<v_{i}{>}_{\text{data}}-<v_{i}{>}_{\text{recon}}\right), \end{array}$$

(8) $$\begin{array}{c} \Delta b_{i}=e\left(<h_{i}{>}_{\text{data}}-<h_{i}{>}_{\text{recon}}\right). \end{array}$$

Among them, *e* is the learning rate, 〈*h*_*i*_〉data is the mathematical expectation defined by the training dataset, and 〈*h*_*i*_〉recon represents the expectation of the model definition after a step of reconstruction.

#### 2.1.4. Basic Structure

**Figure 2:**
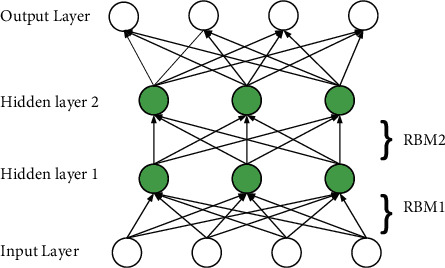
The structure of DBN model.

The deep belief network is composed of multiple restricted Boltzmann machines and a layer of supervised classifiers, as shown in Figure 2. DBN can be summarized into two stages in the whole learning process. the first stage is unsupervised learning, and the second stage is supervised fine-tuning [17, 18].

1. The first stage is to conduct greedy training on multilayer RBM, that is, training layer by layer, and training the model layer by layer from the bottom to the top. The output value of the previous RBM is used as the input value of the next RBM to realize the initialization of the network parameters.
2. The second stage is to fine-tune the model. In order to optimize the objective function of the entire model structure, the BP neural network algorithm or the support vector machine can be used to fine-tune the parameters to achieve global optimization. The fine-tuned network parameters are used as the initial parameters of the entire network. Compared with traditional neural networks, DBN has a higher accuracy rate and at the same time solves the problem of easily falling into local optimality.

### 2.2. Mixed Education Evaluation System

Blended teaching is a new type of education and teaching mode that combines the advantages of traditional face-to-face teaching and online teaching, and integrates information technology with traditional education and teaching in an effective form. The realization of this comprehensive advantage is based on the deep integration of different application methods, different teaching concepts, and different technical means. Blended education teaching has five basic characteristics: (1) the integration of teaching theories; (2) the integration of teaching resources; (3) the integration of teaching environments; (4) the integration of teaching methods; (5) the integration of teaching strategies; and (6) the integration of evaluation methods [19].

Through reading related literature, the teaching evaluation camp covers the entire process of teacher teaching, basically including teaching objectives, teaching design, teaching process, and teaching effect. The concept of “assessing teaching by learning” and the characteristics of a mixed teaching model are constructed in this paper to construct a three-level evaluation index. In order to maintain the consistency of descriptions among indicators at all levels, this article describes the first-level indicators of mixed teaching evaluation in terms of “learning design,” “learning environment,” “learning process,” and “learning effect.”

the above-mentioned first-level index system is used to expand the hybrid education and the teaching evaluation system, the hybrid education and teaching evaluation indicators are expanded, and a hybrid education and the teaching evaluation system are built. The specific evaluation methods are shown in Table 1.

## 3. The Quality Evaluation Model of Hybrid Higher Education Teaching Practice Based on Deep Learning Route

### 3.1. Model Structure

DBN is a generative model based on unsupervised learning, and most of the objects it faces are unknown data, such as nonlinear systems. The main goal of deep learning is to use algorithms to describe changes in data [18]. However, most DBNs build models with dense representations, and there is a big problem with this representation; that is, any fluctuations or noise in the input will greatly change the feature representation vector extracted by the hidden layer, which will lead to the robustness of the network. The rod is poor [21].

This paper introduces an output layer in the top layer of the deep belief network model and implements score level mapping to the characteristics of all colleges and universities' online teaching quality evaluation indicators through the Softmax multiclass classifier based on logistic regression expansion and completes the online teaching quality evaluation of colleges and universities [23]. The online teaching quality score mapping of colleges and universities is a multiclass classification problem, so the extended logistic regression is a Softmax regression. Let {(*x*_1_, *y*_1_),…, (*x*_*n*_,*y*_*n*_)} be the set of the state *x* of each neuron in the hidden layer and the corresponding class label *y* of each neuron; for *M* categories, the corresponding class of the i-th feature for labels *y*_i_∈ {1, 2, ..., *M*}, the *M*-dimensional category probability distribution matrix is as follows:

(9) $$\begin{array}{c} l_{\sigma }\left(x^{i}\right)=\left|\begin{array}{c} p\left(y^{i}=1|x^{i};\sigma \right)\\ p\left(y^{i}=2|x^{i};\sigma \right)\\ \cdots \\ p\left(y^{i}=m|x^{i};\sigma \right) \end{array}\right|=\frac{1}{\sum_{j=1}^{M}e^{\sigma_{J}^{T}x^{i}}}\left|\begin{array}{c} \sigma_{1}^{T}x^{i}\\ \sigma_{2}^{T}x^{i}\\ \cdots \\ \sigma_{M}^{T}x^{i} \end{array}\right|,\\ \sigma =\left|\begin{array}{c} \sigma_{1}^{T}\\ \sigma_{2}^{T}\\ \cdots \\ \sigma_{M}^{T} \end{array}\right|. \end{array}$$

In the above formula, the model parameter matrix of *M* × *N* is *σ*; the input feature dimension is *N*. The expression formula of the cost function of Softmax regression is as follows:

(10) $$\begin{array}{c} J\left(\sigma \right)=\frac{1}{n}\left[\overset{n}{\underset{i=1}{\sum }}\overset{M}{\underset{j=1}{\sum }}1\left\{y^{i}=j\right\}\log\right.\frac{e^{\sigma_{j}^{T}x^{i}}}{\sum_{d=1}^{M}e^{\sigma_{d}^{T}x^{i}}}=\frac{\tau }{2}\overset{M}{\underset{i=1}{\sum }}\overset{N}{\underset{j=1}{\sum }}\sigma_{ij}^{2}. \end{array}$$

**Figure 3:**
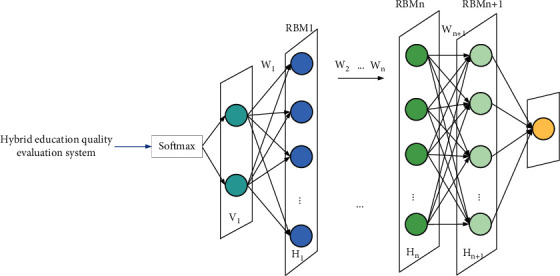
A hybrid university teaching quality evaluation model based on the deep belief network.

The college hybrid teaching quality evaluation system is used as the input value of the deep belief network's online teaching quality evaluation model of colleges and universities. To ensure that the deep belief network model conforms to the characteristics of the input college online teaching quality evaluation system, the first layer of RBM uses Gauss–Bernoulli. RBM is used to map the features entered into the online teaching quality evaluation system of colleges and universities into a binary state. The remaining layers of RBM use Bernoulli-Bernoulli RBM, and the Bernoulli-Bernoulli RBM abstracts the features between the essential relationship. The hybrid higher education teaching quality evaluation model based on the DBN model is shown in Figure 3.

### 3.2. Training Steps

Since the p(*h*_1_;W _1_) of the deep belief network model is consistent with the *p*(*v* | *h*_1_;*W*_1_) of the RBM, there is a greedy layer-by-layer pretraining step of the s-layer hidden deep belief network model in the model.

  Step 1. Train RBM to obtain the weight parameter W1 of the bottom RBM.
  Step 2: Start training the second layer of RBM, initialize the second layer weight parameter *W*2 = W1T to ensure that the deep belief network model with two hidden layers is better than the uninitialized RBM, and then pass fixed W1 to train the second layer of RBM to optimize W2.
  Step 3: Continue to train the third layer of RBM, initialize the third layer RBM weight parameter *W*3 = W2T, train the third layer RBM by fixing W2 to make W3 reach the optimal value, similarly, complete the final layer of RBM weight to reach the optimal value, complete the unsupervised pretraining of the entire deep belief network model.

In the case of optimal learning of the parameters to be tested, for the feature to be tested, and after learning the deep belief network model of the trained model parameters, the probability that this feature belongs to the online teaching quality scores of various colleges and universities is calculated, and the quality score with the highest probability is selected as output result.

## 4. Simulation Experiment

### 4.1. Experimental Data

Taking a university as the evaluation object, the mixed teaching data of the university from March to August of 2020 are randomly selected as the dataset. The data include a total of 5,000 data samples. The online teaching quality evaluation method for colleges and universities based on the deep learning network studied in this paper is used to implement online teaching quality evaluation. The selected evaluation indicators are shown in Table 2. The scoring method of the evaluation indicators is to select 10 experts from outside the school and 5 senior professors familiar with the online teaching situation in the school. The average scoring value is regarded as the final evaluation score of each secondary evaluation index. The interval of this value is (0–1). Each score corresponds to the online teaching quality evaluation level of colleges and universities; that is, the evaluation criteria are shown in Table 3.

### 4.2. Learning and Training Performance

**Figure 4:**
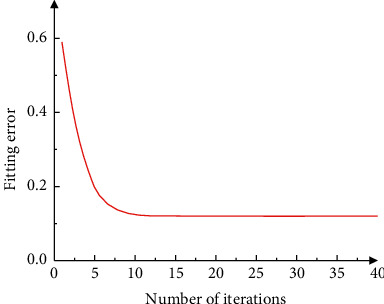
The learning performance and training performance of the DBN model.

In order to test the learning performance and training performance of the DBN model used in this paper, 3,000 data samples out of 5,000 data samples are used as the model training set. The remaining 2,000 data samples are used as the model test set, and the training set sample data are used as the model input, the model training is expanded, and the model fitting error is calculated under different iteration times in the training process. The result is shown in Figure 4. It can be seen from the data in Figure 1 that as the number of iterations increases, the fitting error of the DBN model used in the method in this paper decreases significantly. When the number of iterations reaches 20, the fitting error of the DBN model stabilizes and decreases to below 0.01. The experimental results show that the model used in this method has good learning and training performance, and the fitting error is low.

The 2,000 test samples in the test set are divided into 10 groups and input them into the model for model testing, and the output results of the model are compared with the expected output results. The results are shown in Table 4. From the data in Table 4, it can be seen that the relative error between the test output result and the expected output result after the model is trained is less than 3%, and the evaluation level of the method in this paper is exactly the same as the actual level. Experimental results show that the trained model has evaluation accuracy and can be effectively used for online teaching quality evaluation in colleges and universities.

### 4.3. Evaluate Performance

In order to verify the evaluation performance of the method in this paper, Karl Pearson correlation coefficient and RMSE are used to measure the degree of closeness and fluctuation between the evaluation results of efficient online teaching quality evaluated by this method and the actual teaching quality results. The correlation coefficient *R* and the root mean square error RMSE are counted in the evaluation process of the method in this paper, and the results are shown in Table 5. Analyzing the data in Table 5, we can see that the evaluation correlation coefficients of the method in this paper are all greater than 0.85, and the root mean square error of the evaluation is less than 0.45. The results show that the evaluation results of the method in this paper are highly similar to the actual evaluation level, and the error is small, which can be effectively applied to online teaching quality evaluation in colleges and universities.

Through the above experiments, it can be seen that the method in this paper has good performance in evaluating the quality of online teaching in colleges and universities. For this reason, the method in this paper is used to evaluate the online teaching quality of the colleges and universities. The evaluation results are shown in Table 6. After analyzing the data in Table 6, it can be seen that the method in this paper can realize the quality evaluation of the mixed education of the university by evaluating the various indicators that measure the quality of the mixed education of the university. The mixed education quality of the college is generally evaluated by the method of this article. Colleges and universities can take targeted measures based on the indicators with lower scores.

The evaluation results of this article is to improve the quality of online teaching.

## 5. Conclusion

This paper establishes a hybrid education teaching practice quality evaluation system in colleges and universities, and constructs a hybrid teaching quality evaluation model based on a deep belief network. Karl Pearson correlation coefficient and Root Mean Square Error (RMSE) indicators are used to measure the closeness and fluctuation between the effective online teaching quality evaluation results evaluated by this method and the actual teaching quality results degree. The experimental results show that:

1. As the number of iterations increases, the fitting error of the DBN model decreases significantly. When the number of iterations reaches 20, the fitting error of the DBN model stabilizes and decreases to below 0.01. The experimental results show that the model used in this method has good learning and training performance, and the fitting error is low.
2. The evaluation correlation coefficients are all greater than 0.85, and the root mean square error of the evaluation is less than 0.45, indicating that the evaluation results of this method are similar to the actual evaluation level and have small errors, which can be effectively applied to online teaching quality evaluation in colleges and universities.

## Figures and Tables

**Table 1:** Hybrid higher education teaching quality evaluation system [20].

Evaluation object	First-level index	Second-level index	Third-level index
Blended teaching quality	A1 learning design	B1 learning objectives	C1 learning objectives at different levels of knowledge, abilities, qualities, etc., are positioned accurately and comprehensively, and the online and offline learning objectives are distinct
		B2 learning strategy	C2 learning strategy matches with learning goals
			C3 learning strategies are in line with students' academic conditions
			C4 learning strategies are to meet the learning needs of students
		B3 learning methods	C5 learning methods are diverse and interactive
	A2 learning environment	B4 learning support	C6 learning platform provides guarantee in terms of space, time, and equipment
		B5 learning resources	C7 online learning resources are highly learnable and pertinent
			C8 offline learning resources are more thoughtful and inquiring
			C9 expansion of learning resources is hierarchical and challenging
	A3 learning process	B6 learning links	C10 online and offline learning links are reasonably distributed
			C11 online and offline learning links are closely connected
		B7 learning content	C12 learning content is highly learnable
			C13 online and offline learning content complements
			C14 learning content is cutting-edge and challenging
		B8 learning participation	C15 Student's investment in various teaching links online and offline
			C16 students' thinking and feedback on
		B9 learning assessment	C17 online learning assessment has clear levels
			C18 offline classroom learning assessment is innovative and challenging
	A4 learning effect	B10 learning willingness	C19 increased students' learning enthusiasm and sense of learning achievement
			C20 students' enthusiasm and initiative to explore challenging learning content
		B11 learning ability	C21 increased willingness of students to display their personal learning achievements
			C22 improve students' autonomous learning ability
			C23 students perform well in case analysis, experimental operation, and situational practice
			C24 students can take the initiative to ask questions during the teaching process and propose solutions to the knowledge they have learned
		B12 learning quality	C25, the improvement degree of students' knowledge, ability, quality, and ability matches the learning goals, the goal achievement degree is high, and the students are highly satisfied with the course teaching

**Table 2:** Blended education and teaching evaluation system-first level.

Indicators	Index definition
Learning design	The learning design index refers to the preparatory part of the teacher's preparation for student learning in the mixed teaching process. It guides the development of the entire teaching activity and is the basis of the entire teaching activity. Therefore, it is reflected from the three aspects of learning objectives, learning strategies, and learning methods.
Learning environment	The learning environment index refers to the physical environment in the mixed teaching mode, which provides support for the development of mixed teaching. The influence of this kind of learning environment on the quality and effect of blended teaching cannot be ignored. Therefore, it is reflected from the two aspects of learning support and learning resources.
Learning process	The learning process indicators cover teacher teaching and student learning content, behavior, and assessment in each link of blended teaching. Combining the characteristics of the blended teaching model, this indicator reflects the four aspects of learning links, learning content, learning participation, and learning assessment.
Learning effect	The learning effect index includes two aspects: student learning and teacher teaching. Student learning is reflected in two aspects: willingness to learn and learning ability while teacher teaching is reflected in learning quality.

**Table 3:** Hybrid higher education teaching quality evaluation standard.

Evaluation score	Estimated grade
>0.75	Excellent
0.5∼0.75	Good
0.25∼0.49	Normal
<0.25	Bad

**Table 4:** Model test results.

Dataset	Expected output	Actual grade	Test output	Evaluation level	Relative error (%)
1	0.78	Excellent	0.79	Excellent	1.28
2	0.59	Good	0.58	Good	1.7
3	0.42	Normal	0.43	Normal	2.38
4	0.52	Good	0.53	Good	1.92
5	0.11	Bad	0.11	Bad	0
6	0.24	Bad	0.24	Bad	0
7	0.88	Excellent	0.87	Excellent	1.14
8	0.46	Normal	0.47	Normal	2.17
9	0.82	Excellent	0.81	Excellent	1.22
10	0.65	Good	0.66	Good	1.54

**Table 5:** *R* and RMSE statistical results.

Dataset	*R*	RMSE
1	0.92	0.25
2	0.88	0.33
3	0.95	0.41
4	0.89	0.45
5	0.92	0.28
6	0.95	0.17
7	0.89	0.41
8	0.86	0.22
9	0.87	0.34
10	0.96	0.26

**Table 6:** Quality evaluation results of higher mixed education.

First-level index		Second-level index		Evaluation result	
Number	Weight	Number	Weight		
A1	0.2	B1	0.4	0.75
		B2	0.5	0.85
		B3	0.1	0.74	0.72
A2	0.2	B4	0.5	0.79	
		B5	0.5	0.72	
A3	0.4	B6	0.2	0.56	
		B7	0.3	0.71	
		B8	0.3	0.69	
		B9	0.2	0.75	
A4	0.2	B10	0.3	0.46	
		B11	0.4	0.62	
		B12	0.3	0.71	

## Data Availability

The dataset can be accessed upon request.
